# Will our cardiomyopathy patients accept gene therapy?

**DOI:** 10.1007/s12471-022-01665-z

**Published:** 2022-03-02

**Authors:** P. A. Doevendans, C. Kupatt, M. Giacca, P. Glijnis

**Affiliations:** 1 Department of Cardiology, University Medical Centre Utrecht, Utrecht, The Netherlands; 2 Netherlands Heart Institute, Utrecht, The Netherlands; 3 Medizinische Klinik und Poliklinik I, University Clinic rechts der Isar, TUM Munich, Munich, Germany; 4 School of Cardiovascular Medicine & Sciences, King’s College London, London, UK; 5 PLN Foundation, Middenmeer, The Netherlands

**Keywords:** Gene editing, Cardiomyopathy, Viral vectors

## Abstract

Novel techniques such as gene therapy are becoming available in an attempt to cure inherited diseases. Before these new therapies can be offered to patients, we need to be aware of potential reservations or objections, not only from patients and their surroundings but also from the public. In addition, legal issues and costs need attention before curative gene therapy can be applied in the clinic. As this therapeutic approach is closer to becoming a reality, now is the right time to start the debate.

We are moving very close to an incomplete but effective cure for genetic diseases, as shown by the first results of in vivo gene editing in a progeria mouse model [1]. After injection of an adeno-associated viral (AAV) vector, a mutation in the gene for nuclear lamin A (*LMNA*) was corrected in homozygous mice. Although the effectiveness of the correction varied from 20% to 60% in different organs, the lifespan of the mice increased from 7 to 18 months (normal average lifespan is 24 months). The production of progerin, a toxic protein, was markedly reduced [1]. If these results could be reproduced in man, this could more than double the life expectancy of progeria patients, from 14 to over 30 years. The fact that children develop this disease makes the ethical discussion even more complex. Of importance is that the public opinion may differ from that of patient advocacy groups.

On the website of the National Institutes of Health (NIH) in the United States, we found the following statement: ‘The Progeria Research Foundation was thrilled to collaborate on this seminal study with Dr. Collins’s group at the NIH and Dr. Liu’s group at Broad Institute,’ said Leslie Gordon, MD, PhD, who is a coauthor of the mentioned paper and the medical director of The Progeria Research Foundation, which partially funded the study. ‘These study results present an exciting new pathway for investigation into new treatments and the cure for children with progeria’ [2].

Another breakthrough was reported by Moretti et al. in models for Duchenne muscular dystrophy, which primarily affects boys. Although treatment with an AAV vector could again not induce a complete nor a perfect repair, the shortened dystrophin protein appeared to be effective in skeletal muscle, the diaphragm and the heart to improve muscle function [3].

The first ocular gene therapy that was approved by the American Food and Drug Administration, voretigene neparvovec-rzyl (Luxturna; Spark Therapeutics, USA), uses an AAV2 vector to deliver a functional copy of the *RPE65* gene for the treatment of Leber congenital amaurosis type 2. This autosomal recessive inherited eye disease leads to impaired vision or even blindness at birth, but the phenotype is variable. Clinical trials to evaluate safety and effectiveness are ongoing, [4] supported by the Foundation Fighting Blindness (www.fightingblindness.org). While this therapy does not provide a cure, it leads to prolonged normal protein expression. In Australia, a protocol has been approved to evaluate the ethics of ocular gene therapy [5]. The research group involved is in the process of developing a new ‘Attitudes to Gene Therapy for the Eye’ tool.

These papers are crucial in several ways and should ignite discussion on multiple levels. First, they show that DNA repair of a specific mutation is feasible in a living organism without overt side effects. Furthermore, although the affections involve systemic diseases, an incomplete gene correction based on one injection had a major effect on protein function and/or survival. This indicates that the therapy designed and applied does not have to be perfect (100% correction) to be effective. In addition, *LMNA* is a well-known gene in cardiovascular disease and is linked to dilated cardiomyopathy and arrhythmias. At the same time, Duchenne patients often develop dilated cardiomyopathy. Moreover, alternative gene therapies have reached the clinic, for instance for eye diseases.

We also need discussion with the patients and their relatives, the public and healthcare providers. If we are not able to finetune discussions, we may be creating treatment strategies that patients do not want to accept. One key issue are the costs involved in these therapies, especially as curative gene therapy needs a customised approach depending on the mutation. In the general public, we recently observed some surprising reactions and resistance to vaccinations, which some believe alter human DNA based on some layman’s tale or noninformation.

The University Medical Centre Utrecht (UMC Utrecht), in collaboration with the Leiden University Medical Centre (LUMC) and Amsterdam University Medical Centres (Amsterdam UMC), a grant was obtained from the Dutch Research Council (*Nederlandse Organisatie voor Wetenschappelijk Onderzoek*) to further develop both AAV and adenoviral vectors. In addition, a PSIDER grant has been awarded to the Hubrecht Institute (located in the Netherlands), UMC Utrecht, LUMC and Amsterdam UMC for developing prime editing of *LMNA* and the Dutch phospholamban/PLNR14Del-related cardiomyopathies (principal investigator of PSIDER-Heart is Eva van Rooij).

In the PSIDER grant, a substantial budget is allocated to the ethical evaluation of the impact of gene therapy. In collaboration with the PLN Foundation (https://en.plnheart.org), we are planning to create five documentaries to facilitate national and international discussion on gene therapy. One of the topics of the recently launched Horizon Europe programme is ‘Next-generation advanced therapies to treat prevalent and high-burden diseases with unmet needs’. This novel programme puts a stronger accent on impact than the previous Horizon 2020 funding programmes. We are planning to combine innovative strategies in this application to further improve the efficacy of gene editing, large-animal research and ex vivo and in vivo perfusion models.

In the next 3–4 years, we have to define the optimal strategy to deliver gene therapy to a sufficient number of cardiomyocytes and show improvement of contractile function, preferably without inducing arrhythmias. When only the heart needs treatment, we may have to develop an in situ isolated cardiac perfusion system if systemic applications are not effective enough. We will learn how to improve the treatment in small and large humanised animal models. Next, we have to decide what form of therapy is acceptable for which patient and if it is still possible to improve function in advanced heart disease. In the end, we may have a solution for patients with little or no symptoms and limited motivation for gene therapy but nothing to offer highly motivated patients with end-stage cardiac failure. An important issue will be the costs, as healthcare expenses could become limited, and legal issues. The European Parliament should also check and update its existing legislation on the use of gene therapy.

It is clear to us that we are entering a new era with therapeutic options for patients with unmet needs. We at the Netherlands Heart Institute hope to be able to alter the course of inherited cardiac diseases together with our patients through biological innovations.
